# A Deep Learning-Based Model Approach for Quantitative Analysis of Cell Chemotaxis in a Microfluidic Chip

**DOI:** 10.3390/s25113515

**Published:** 2025-06-03

**Authors:** Hongxuan Wu, Fei Zhang, Mingji Wei

**Affiliations:** School of Electrical and Information Engineering, Jiangsu University, Zhenjiang 212013, China; wuhongxuan@ujs.edu.cn (H.W.); 2111907013@stmail.ujs.edu.cn (F.Z.)

**Keywords:** cell chemotaxis, deep learning, quantitative analysis, microfluidic chip

## Abstract

The rapid and accurate quantitative analysis of cell chemotaxis, which is essential in biology, medicine, and drug development, enables the evaluation of the directional migration capability of cells and the simulation of in vivo cell chemotaxis. However, traditional methods for studying cell chemotaxis often depend on complex experimental procedures, which are not only time-consuming and labor-intensive but also prone to human error. Recently, the rapid advancement of microfluidic technology and deep learning has provided a new way for evaluation of cell chemotaxis. In this study, a chemotaxis evaluation method based on microfluidics and deep learning is proposed. A microfluidic device was designed to simulate cell chemotaxis, allowing for the controlled assessment of cell chemotaxis by generating chemical gradients within microchannels and shear stress. Concurrently, deep learning technology was introduced to identify the migrated and non-migrated states of cell images, thereby enabling the automatic counting and analysis of chemotactic cells. Compared with traditional manual assays, this method not only reduced time and labor costs but also achieved higher accuracy and reproducibility. This innovative approach, which integrates microfluidics and deep learning, provides a novel perspective and tool for cell chemotaxis research. This method not only offers a fresh perspective on cell migration analysis but also has the potential to significantly advance the field of biomedical research, particularly in biosensor development related to drug discovery and disease diagnosis.

## 1. Introduction

Cell chemotaxis refers to the ability of cells to migrate directionally in response to biological and physiological gradients [1]. This movement is typically triggered by exogenous chemicals, growth factors, cytokines, or other molecular gradients [2]. Cell chemotaxis plays a crucial role in various physiological and pathological processes within organisms, including development, immune responses, wound healing, and tumor metastasis [3].

Quantitative evaluation of cell chemotaxis primarily depends on devices that can establish stable chemokine concentration gradients and on methods for assessing cell chemotaxis [4]. Traditional gradient generators, such as the Boyden chamber [5] and the Transwell chamber [6], are widely used. These chambers typically consist of two adjacent compartments separated by a porous membrane, relying on cells migrating through the membrane towards a chemotactic substance in the lower chamber, with quantification usually achieved by counting migrated cells. To realize the real-time observation in Boyden chambers, devices like the Zigmond chamber [7], Dunn chamber [8], and Insall chamber [9] were developed. These allow observation of cell movement through a gradient placed on a glass surface [10]. However, these devices critically rely on simple diffusion to generate gradients [11], which is largely manual, making the process time-consuming, labor-intensive, and prone to error. More critically, the static culture conditions in these chambers cannot fully replicate the complex microenvironment [12] and microscale dynamics of cells within the human body, leading to an incomplete simulation of gradient-induced cellular responses. Given these limitations, there is an urgent need for improved systems capable of dynamically generating chemical concentration gradients to explore true cell chemotaxis processes more accurately.

In recent years, microfluidic chip technology has emerged as a powerful platform in biomedical engineering [13] (e.g., drug screening [14], chemical synthesis [15], cell chemotaxis [16] and cell apoptosis [17] etc.), providing a novel means to dynamically generate chemical concentration gradients [18]. Microfluidic technology overcomes the limitations of conventional methods by enabling precise liquid control at the micron-scale and allowing accurate mimicry of specific chemical concentration gradient stimulation in cell microenvironments [19,20]. Jan Schwarz et al. focused on developing a microfluidic chamber capable of measuring cell chemotaxis responses to surface immobilized and soluble molecular gradients, particularly chemokines [21]. Sebastien et al. described a microfluidic chip capable of generating orthogonal linear concentration gradients by embedding cells in a 3D scaffold [22]. Campbell et al. designed a planar microfluidic network device to generate concentration gradients with the shape of any given monotonic function [23]. More recently, advancements continue to refine microfluidic gradient generation. For instance, Ding Laiqian et al. demonstrated a multi-channel microfluidic system to study the response of tumor cells to different oxygen gradient conditions [24]. Huang Sisi proposed a capillary-driven embedded obstacle-type micro-mixer–concentration gradient generator [25]. So, the microfluidic chip can establish stable and different profile gradients in flow-based environments [26]. Collectively, these microfluidic platforms can establish stable gradients with diverse profiles in flow-based environments, simulating the in vivo microenvironment while offering the characteristics of fast, accurate analysis and low consumption, providing robust device support for in-depth chemotaxis research [27].

Beyond the need for advanced gradient generation devices, there remains an urgent need for rapid and accurate methods to evaluate cell chemotaxis. Traditional evaluation methods of cell chemotaxis research mainly used microscopic observation and image processing technology [28]. Although these methods can achieve quantitative analysis of cell chemotaxis to a certain extent, the traditional image-processing algorithmsare still limited by complex operations, time consumption, and low accuracy [29]. Therefore, it is of great practical significance to develop an efficient and accurate method for quantitative evaluation of cell chemotaxis. The rapid development of artificial intelligence (AI) and deep learning (DL) has provided new solutions to improve accuracy. DL (e.g., the transformer model, Yolo model, and ResNet model, etc.) can automatically extract features from a large number of microscope images to achieve efficient analysis of cell chemotaxis [30]. Compared with traditional manual image processing, it allows automated analysis of cell chemotaxis, which greatly improves data processing speed [31]. Crucially, DL can be applied to extract key information and accurately detect migrated cells [32], effectively improving the accuracy of quantitative analysis of cell chemotaxis.

In this study, we developed a novel method to evaluate cancer cell chemotaxis by integrating microfluidic technology with advanced deep learning. A microfluidic chip was designed to generate concentration gradients and shear stress, simulating the in vivo chemical environment to induce chemotactic responses in cells. To enhance the accuracy of migrated cell detection, the Yolo model was improved, introducing the DCSPP-Yolo framework with an attention mechanism. This modification allows the model to more effectively distinguish between migrated and non-migrated cells. We conducted chemotaxis experiments using the microfluidic chip, capturing images of both migrated and non-migrated cells. The performance of our DCSPP-Yolo model was validated, achieving a precision rate of 96.56% and a recall rate of 96.51%, significantly outperforming traditional Yolov5 models. Furthermore, a comparative analysis demonstrated that our method offers superior accuracy and efficiency compared to conventional approaches. The deep learning-based detection method showed a high correlation with manual counting (R^2^ = 0.98), confirming its reliability for precise and rapid identification of migrated cancer cells within microfluidic systems.

## 2. Materials and Methods

### 2.1. Cell Culture

Human breast cancer cells (MCF-7), obtained from Jiangsu University Medical School in China, were cultured in Dulbecco’s Modified Eagle’s Medium (DMEM) supplemented with 10% fetal bovine serum (FBS) from Gibco Gibco (Grand Island, NY, USA). To optimize cell adhesion, the microchannel chip was pre-incubated at 37 °C with 5% CO_2_ in a a water jacketed CO_2_ incubator (Heal Force^®^ (Shanghai, China)) before experiments. The microchannel substrate was coated with fibronectin (30 μg/mL) for 2 h to enhance cell adhesion, followed by thorough washing with phosphate-buffered saline (PBS) using a syringe pump from Baoding Shenchen Precision Pump Co., Ltd. (Hebei, China).

Cell dissociation was performed using trypsin-EDTA from Life Technologies GmbH (Darmstadt, Germany) for 2 min. After centrifugation at 600 rpm for 5 min, the supernatant was discarded. The remaining cell pellet was resuspended in 3 mL of fresh DMEM with 10% FBS by gentle agitation. Cell suspensions, at a density of 2 × 10^5^ cells/mL, were introduced into the microfluidic channels through polytetrafluoroethylene (PTFE) tubes using a syringe pump.

### 2.2. Design and Function the Microfluidic Chip

**Figure 1:**
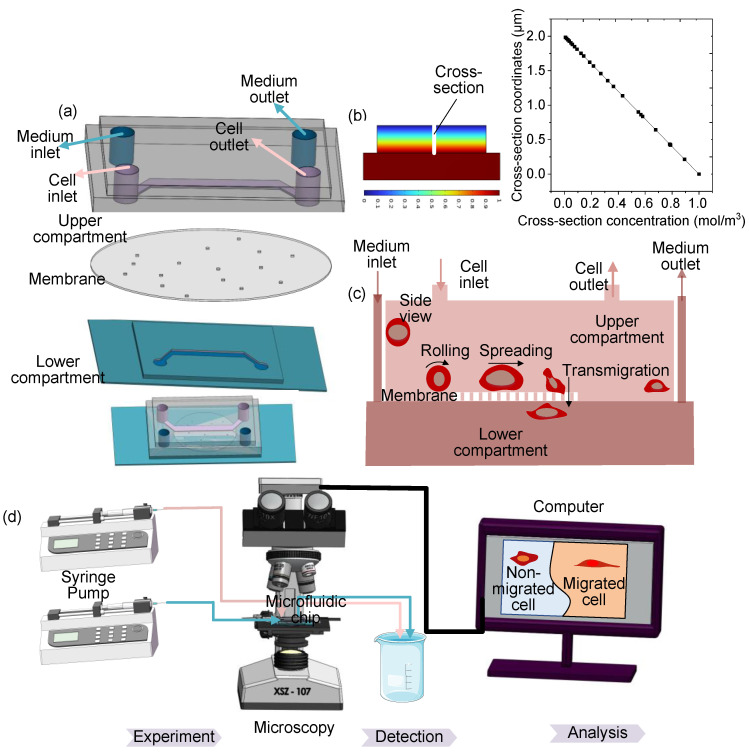
Cell chemotaxis experiment. (**a**) Exploded view and overall schematic diagram of the three-layer structure of the microfluidic chip, showing the upper and lower PDMS compartments and the polycarbonate membrane in between. (**b**) Simulation for gradient profile. (**c**) Prinple schematic diagram of cell chemotaxis assay after cell inoculation. (**d**) Overall process of cell chemotaxis study.

An integrated microfluidic chip was designed (Figure 1a) to quantitatively analyze the cell chemotaxis. The microfluidic chip comprises three principal components: the upper compartment, the membrane, and the lower compartment. The chip was fabricated from polydimethylsiloxane (PDMS), chosen for its transparency, durability, and flexibility [33]. Two PDMS substrates, patterned with grooves using photolithography, form the upper and lower compartments. Inlet and outlet holes were incorporated into a transparent solid layer.

A polycarbonate membrane, designed with a porous structure, acts as a physical barrier between the upper and lower compartments to facilitate cell chemotaxis assays. The membrane featured uniformly distributed cylindrical pores with a diameter of 8 μm and a pore density of approximately 1 × 10^5^ pores/cm^2^. The pore diameter of 8 μm was selected to be sufficiently large to permit the transmigration of target cells (e.g., neutrophils or cancer cells) while small enough to prevent significant convective flow or passive cell settling between compartments, thereby ensuring that cell movement across the membrane is predominantly driven by chemotaxis [34]. The pores were fabricated using a track-etching technique to ensure high precision and uniformity. The membrane was 10 μm thick, and the pores were vertically aligned in a regular configuration, allowing for consistent transmigration of cells during the assay. The components are assembled through plasma bonding, with the lower compartment also bonded to a glass slide. High-energy oxygen plasma treatment enhances the wettability of the PDMS surface, ensuring effective bonding with other surfaces [35]. Plasma bonding is also used to attach the porous membrane to both the upper and lower compartments, establishing communication between the inlet and outlet channels. This configuration allows for the generation of stable chemical gradients across the membrane. A chemoattractant solution is introduced into the lower compartment while cell suspension and medium/buffer are maintained in the upper compartment. Diffusion through the pores establishes a linear concentration gradient perpendicular to the membrane surface over time, driving directional cell migration. This fabrication process results in a functional microfluidic system for controlled cell culture and migration studies.

The final microfluidic chip features a rectangular cross-sectional microchannel (250 μm in height, 1 mm in width) and a channel length of 1 cm (from inlet to outlet). The inlet and outlet ports have a diameter of 1 mm, facilitating easy fluid injection and collection. The porous membrane has a thickness of 10 μm and contains numerous small pores, each 8 μm in diameter. The overall schematic of the microfluidic chip, including the inlet and outlet, is shown in Figure 1a.

The generated chemokine gradient profile was computationally validated using COMSOL Multiphysics ^®^6.3 software (Figure 1b). A 2D model incorporating the chip geometry, laminar flow, and species transport physics confirmed the establishment of a stable, near-linear transmembrane concentration gradient between the chemokine-rich lower compartment and chemokine-free upper compartment under operational flow rates.

### 2.3. Chemotaxis Assays

Cell chemotaxis assays were conducted utilizing the microfluidic chip to scrutinize cellular behavior in response to chemical gradients. All migration assays were performed with MCF-7 cells under serum-reduced conditions (10% FBS) during 10–12 h period. MCF-7 cells have a typical doubling time of 24–30 h; in preliminary studies under identical conditions, not many cells entered mitosis during a 12 h window. Therefore, while a small fraction of new cells could arise via division, this is unlikely to have significantly affected the total counts of migrated versus non-migrated cells. As illustrated in Figure 1c, the schematic diagram provides a clear depiction of the core principles underlying chemotaxis research. It showcases the establishment of a chemical concentration gradient between the upper and lower compartments of the microfluidic device, which serves as the driving force for cancer cells to undergo migration. This migration is facilitated by a series of cellular processes: initial adhesion to the substrate, subsequent cellular extension, and ultimately, transmigration through the pores of the membrane that separates the two compartments [36]. This coordinated movement is a direct response to the chemical gradient. Figure 1d shows an overview of the cell chemotaxis assay. Commencing the experiment, cells and CCL19 chemokine (PeproTech, Cranbury, NJ, USA) were introduced concurrently into the microfluidic channels through discrete inlets, facilitated by syringe pumps from Baoding Shenchen Precision Pump Co., Ltd. CCL19, a member of the C-C motif chemokine ligand family, plays a key role in immune cell trafficking. It binds to the CCR7 receptor and induces directional migration of various cells, including T lymphocytes and dendritic cells. In this study, CCL19 was used as a representative chemokine to simulate a chemical gradient and investigate the chemotactic response of cancer cells. The migration of cells, including both migrated and non-migrated populations, was monitored using an inverted phase-contrast microscope (Olympus CKX53, Japan) equipped with a 20× objective lens. Optical images were recorded with a digital single-lens reflex (DSLR) camera (Canon EOS 5D Mark III, 5760 × 3840 pixels) connected via a C-mount adapter, and acquired using Canon EOS Utility 3.0 software. The recorded images were processed and analyzed using a custom deep learning model implemented in Python 3.8 with TensorFlow 2.6. Then, the migrated and non-migrated cells were identified, and the chemotaxis ability (*CA*) was evaluated by the number rate according to the Equation (1).

(1) $$CA=\frac{N_{\mathrm{migrated}}}{N_{\mathrm{migrated}}+N_{\mathrm{non}-\mathrm{migrated}}}$$

where *N*_migrated_ is the number of cells that migrated through the pore, and *N*_non-migrated_ is the number of the non-migrated cells.

### 2.4. Design of the Deep Learning Model

With the rapid development of deep learning, Convolutional Neural Network (CNN) models have been widely applied in various fields, particularly in image classification recognition and target localization detection [37]. CNNs, a specialized type of multilayer perceptron (MLP) or feedforward neural network, typically consist of input layers, convolutional layers, pooling layers, fully connected layers, and output layers [38].

Our main goal is to classify and recognize migrated and non-migrated cells to accurately quantify cell chemotaxis. To achieve this, CNN models are utilized. Among many CNN models, AlexNet [39], VGG [40], ResNet [41], and Yolo models are notable [42]. Yolo, in particular, is a real-time object detection algorithm celebrated for its ability to scan and detect objects across an entire image in a single pass, thereby eliminating the need for multiple scans [43].

**Figure 2:**
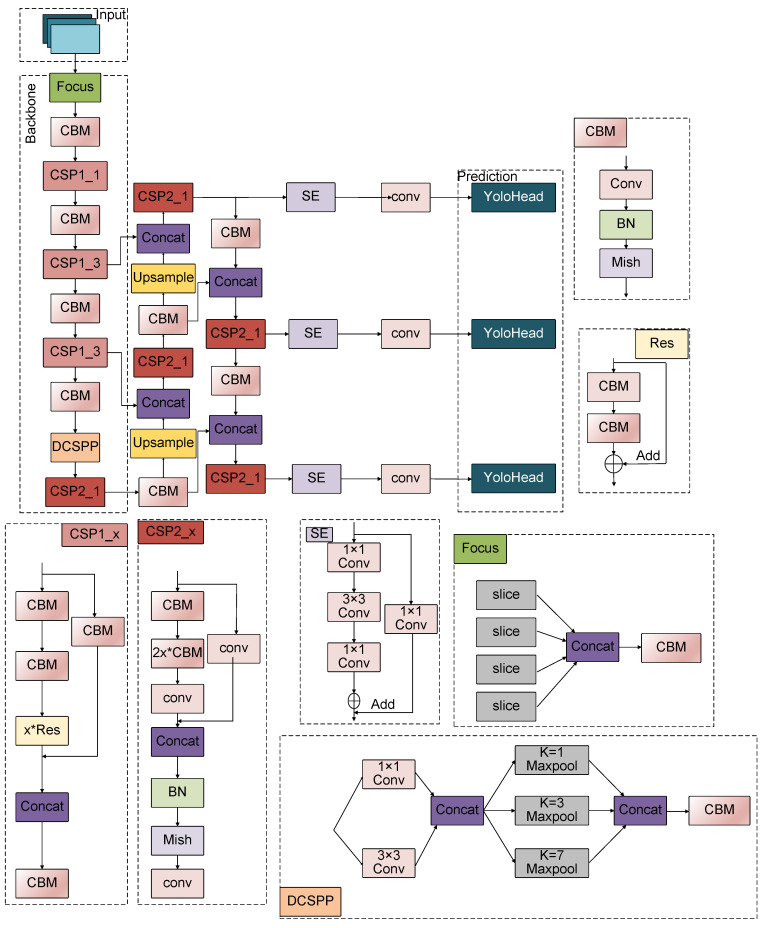
DCSPP-Yolo deep learning model architecture used for identification and counting of migrated cells.

The success of deep learning hinges on the availability of accurate training data and an effective neural network structure [44]. To address the unique challenges of cell chemotaxis experiments, we propose an improved Yolo model specifically tailored for small-target detection at the micron level, which call DCSPP-Yolo. Small-target recognition in the original network is often inadequate for this context due to the microscopic size of cells [45]. Our innovative approach introduces a small-target detection method based on the CSPDarknet53 model, as illustrated in Figure 2. The DCSPP-Yolo model architecture comprises an input layer, a feature extraction network (backbone), a feature fusion network (neck), and an output detection head (prediction head).

A key innovation of this work is the introduction of the DCSPP module, which significantly improves over the traditional SPPF module. Unlike the SPPF module, which reduces dimensionality directly through a convolution layer followed by pooling operations, the DCSPP module leverages convolution kernels of varying scales to capture larger receptive field information, enhancing precision.

Additionally, the SE (Squeeze-and-Excitation) attention mechanism was integrated into the neck network layer of the DCSPP-Yolo model. This integration strengthens the model’s feature extraction capability, enabling it to accurately locate and count migrated cells in complex microfluidic chip environments. Furthermore, to enhance cell counting accuracy, a balancing coefficient in the loss function was introduced, which was defined as follows:

(2) $$L_{\mathrm{loss}}=\lambda_{1}L_{cls}+\lambda_{2}L_{obj}+\lambda_{3}L_{loc}$$

Among them, *λ*_1_, *λ*_2_, and *λ*_3_ are the balance coefficients, and *L_obj_* is the confidence loss. The confidence level is used to represent the credibility of the prediction box, and the prediction boxes that may contain targets are screened. *L_loc_* is the positioning loss, which is used to calculate the degree of overlap between the predicted box and the real box. *L_cls_* is the classification loss, used to determine whether the predicted box category is consistent with the label category.

We optimized the post-processing steps of the model and introduced the non-maximum suppression (NMS) strategy, which can effectively eliminate overlapping detection results and retain the most representative cell bounding boxes. This avoids repeated counting of the same cells, thereby improving counting accuracy and stability [46].

In addition, the model performance indicators for cell detection and recognition mainly include accuracy, precision, recall [47]. The calculation formula is as follows:

(3) $$\begin{array}{l} accuracy=\frac{TP+TN}{TP+TN+FN+FP}\\ precision=\frac{TP}{TP+FP}\\ recall=\frac{TP}{TP+FN} \end{array}$$

*TP* indicates that the model correctly detected the cell and positioned it within the bounding box. *FP* means that the model incorrectly misidentifies irrelevant regions (such as noise or background) as cells, resulting in additional bounding boxes. *FN* means that the model failed to detect the actual cells, resulting in the cells not being positioned within the bounding box.

## 3. Results and Discussion

### 3.1. Influence Factor of Shear Stress and Cell Density

In our study, the regulatory effects of shear force, cell density, culture time, and chemokine concentration on cell chemotaxis were comprehensively investigated [48]. These factors collectively form a complex regulatory network that influences dynamic cell chemotaxis.

**Figure 3:**
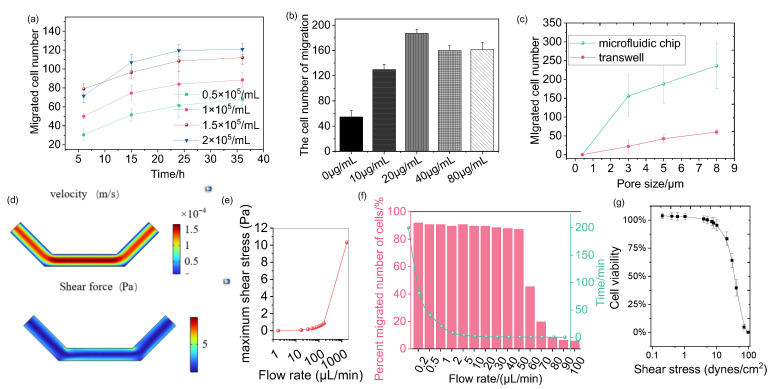
Optimization of microfluidic chemotaxis assay parameters. (**a**) Effect of cell density and culture time on cell migration (**b**) Chemotaxis response of MCF-7 cells to varying CCL chemokine concentrations. (**c**) Effect of membrane pore size on cells. (**d**) COMSOL simulation results of the velocity and shear stress within the lower chemokine channel. (**e**) Calculation profile showing the maximum shear stress experienced by cells in the lower channel. (**f**) Effect of chemokine flow rate and the injection time on chemotaxis efficiency. (**g**) Evaluation of the impact of flow-induced shear stress on cell viability.

Figure 3a illustrates the effects of cell density and culture time on cell chemotaxis. At a cell density of 0.5 × 10^5^ cells/mL, the lowest number of migrated cells was observed, which may be attributed to reduced cell adhesion. In contrast, the highest number of migrated cells was recorded at a density of 2 × 10^5^ cells/mL, suggesting that higher cell densities may enhance cell interactions or other factors promoting migration. The trends observed in the graph indicate that the number of migrating cells increased gradually over time, suggesting that allowing a longer culture period enables more cells to respond to the chemokine gradient and migrate.

The effect of CCL chemokine concentration on cell chemotaxis was evaluated by counting the number of migrated cells. The results reveal a complex concentration dependence, as illustrated in Figure 3b. At lower concentrations of CCL, the number of cells gradually increased, indicating that CCL positively influences cell guidance and proliferation. This increase suggests that low CCL concentrations attract cells, thereby promoting their migration and proliferation. Interestingly, the cell number reached a maximum at a CCL concentration of 20 µg/mL, which may represent an optimal concentration for providing the best survival conditions or guiding the most efficient migration process. However, as CCL concentrations increased to 40 µg/mL and 80 µg/mL, the cell number decreased, indicating an inhibitory effect due to excessive concentrations. This decline suggests a negative cellular response at high CCL levels, potentially involving apoptosis or other adverse effects.

Additionally, the pore size of the microfluidic chip membrane significantly impacts cell chemotaxis. The effect of varying pore sizes was studied, as shown in Figure 3c. For cells with a size of 3 µm, migration was relatively limited, and cell deformation was unnecessary. This limitation occurs because the cells, being relatively large compared to the pore size, fall directly through the pores into the lower chamber, preventing effective attachment to the membrane surface. Therefore, the pore size directly constrains cell chemotaxis in microfluidic systems. As cell size increased to 5 and 8 µm, cell chemotaxis also increased, with deformation observed in cells sized at 8 µm. This indicates that larger cells must deform to pass through the pores, allowing them to attach to the membrane surface and promoting efficient migration. Such deformability is crucial for navigating complex environments where cells must adjust their shape to pass through restricted spaces. These findings align with traditional transwell experiments, highlighting the ability of microfluidic systems to replicate cell behavior observed in conventional assays.

Moreover, microfluidic chips facilitate a higher number of migrated cells compared to traditional chamber assays. Figure 3c shows that, at cell sizes of 5 µm and 8 µm, chemotactic ability is significantly enhanced. This suggests that microfluidic systems improve migration efficiency, primarily due to their ability to accommodate various pore sizes and support cell deformability. Thus, microfluidic platforms provide a more reliable and detailed environment for studying cell chemotaxis and chemotaxis than traditional methods.

In the microfluidic system, the impact of varying flow rates on cell behavior was investigated. Figure 3d simulates the velocity distribution and corresponding shear stress in the lower microfluidic channel. The simulation results reveal that velocity gradients are most pronounced near the channel walls, where the flow interacts with the surface, and gradually decrease toward the center. These velocity gradients generate a spatially heterogeneous shear stress distribution, with the highest shear stress observed near the walls. Shear stress, also called shear force, is the mechanical force exerted by a fluid on the surface of a cell and is calculated as follows:

(4) $$\tau =\mu \frac{du}{dy}$$

where τ is shear stress, μ is the fluid’s viscosity, and $\frac{du}{dy}$ is the shear rate.

Figure 3e quantitatively shows the relationship between maximum shear stress and flow rate, revealing a positive correlation. At flow rates ranging from 0 to 100 µL/min, the maximum shear stress remains relatively low, generally below the threshold that cells can tolerate, thus having limited effects on cell morphology and migration. However, when the flow rate increases to 1000 µL/min, the maximum shear stress rises significantly, reaching 10 Pa, which exceeds the physiological tolerance of the cells. This high shear stress causes considerable mechanical damage to the cells, including morphological distortion and disruption of adhesion, potentially inducing apoptosis [49]. The experimental observations in Figure 3f align with these simulations, showing the impact of flow rates on cell chemotaxis. At flow rates of 0–50 µL/min, the number of migrating cells remains stable, indicating that cells can adapt to mild shear forces and maintain normal migratory activity. However, a sharp decline in cell chemotaxis is observed at higher flow rates (80–100 µL/min), correlating with the increase in maximum shear stress.

To directly assess the impact of flow-induced shear stress on cell viability, live/dead staining (Calcein-AM/PI) was performed on cells exposed to the respective flow rates for 12 h (Figure 3g). Consistently, cell viability remained high (>90%) at flow rates of 0.1–1 dyne/cm^2^. In contrast, a significant increase in cell death was observed at 10 dyne/cm^2^ (approximately 75% cell viability) and 100 dyne/cm^2^ (approximately 0% cell viability). This direct quantification of cell death strongly supports that the drastic reduction in cell chemotaxis at high flow rates (Figure 3f) is primarily attributable to flow-induced cytotoxicity and loss of cell viability, rather than merely an inhibition of migratory function.

Thus, the effect of flow rate on cell chemotaxis is significant because shear stress influences cell behavior, morphology, and viability. An injection flow rate of 50 µL/min is therefore selected as a balanced condition, providing a compromise that accurately simulates physiological conditions while minimizing excessive mechanical stress on the cells.

### 3.2. Experimental Analysis of the Improved Model and Parameter Optimization Analysis

The chemotaxis database used in this study was constructed from real experimental data collected on a microfluidic platform for cancer cell migration. Microfluidic chips generated chemical gradients to induce migration, and images were acquired during the chemotaxis assays. Data were processed on a computer with an Intel Core i9-10900K CPU running Windows 10 Professional. The LabelMe annotation tool was used to label migrated and non-migrated cells.

Three experiments were conducted, collecting 500 cell image samples. Due to stringent experimental conditions and the high precision required by the microfluidic platform, the sample size was limited. In addition, some images were excluded due to poor quality or unclear cell morphology. To address the limited sample size, data augmentation techniques were applied. Using random flipping, small-angle rotation, and brightness adjustment, the original 500 samples were expanded to approximately 2000 samples, enhancing the adaptability of the model. The augmented dataset was divided into training, validation, and test sets in a 7:1:2 ratio, with 1400 samples for training, 180 for validation, and 420 for testing. This allocation ensures effective model evaluation and optimization.

**Figure 4:**
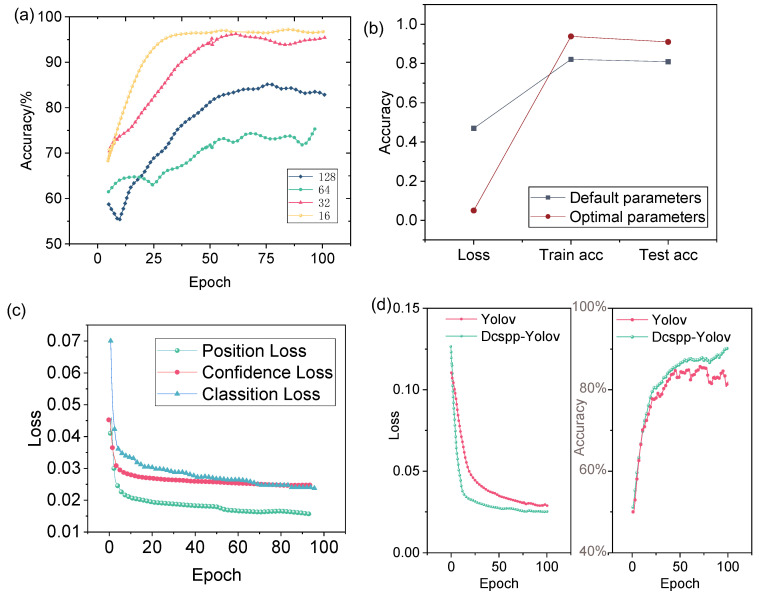
Training and performance evaluation of the DCSPP-Yolo model. (**a**) Accuracy rates corresponding to different batch sizes. (**b**) Comparison of training convergence and performance using unoptimized vs. optimized hyperparameter values. (**c**) Position loss, confidence loss, and classification loss curves during DCSPP-Yolo model training. (**d**) Comparison of metrics between the DCSPP-Yolo model and the traditional Yolo model.

Based on the CSPDarknet53 model, an improved multi-scale experiment called DCSPP-Yolo was developed. Hyperparameters like learning rate and batch size significantly affect performance [50]. To improve the accuracy and efficiency of the DCSPP-Yolo model, different parameter settings are trained. The accuracy rates corresponding to different learning rates are shown in Figure 4a. As can be observed, the performance of the model is improved with specific learning rates, indicating the sensitivity of the DCSPP-Yolo model to this hyperparameter. An optimal batch size of 16 is identified through experimentation, leading to faster convergence and higher accuracy in detecting cancer cell migration. In addition, the learning rate, epoch, etc. are optimized, as shown in Table 1. The impact of unadjusted parameter values versus optimized parameter values on model training is shown in Figure 4b. The results clearly highlight the differences in training and testing accuracy, with optimized parameters leading to lower loss.

After the parameter optimization analysis, the improved DCSPP-Yolo model was trained and further evaluated. As shown in Figure 4c, the loss values of the DCSPP-Yolo model are presented for different training epochs. It is evident that, as training progresses, the loss values steadily decrease, indicating that the model is converging and that the optimized hyperparameters contribute to better data fitting. Additionally, the comparison between the DCSPP-Yolo model and the traditional Yolo model, as illustrated in Figure 4d, further highlights the benefits of the improved DCSPP-Yolo. The traditional Yolo model exhibited relatively higher error rates and lower accuracy, suggesting that the optimization of the DCSPP-Yolo model not only improved its performance in detecting cancer cell migration but also made it more robust to variations in the dataset.

### 3.3. Image Analysis of Cell Chemotaxis Assays

**Figure 5:**
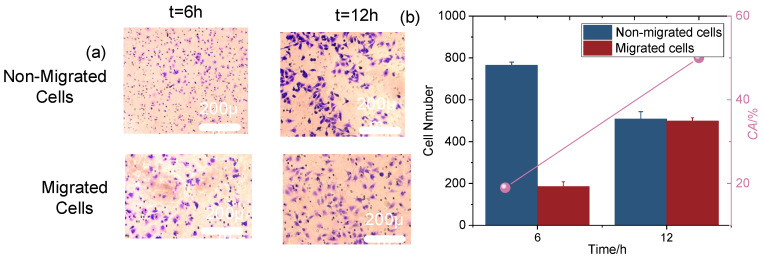
Microscopic analysis and quantification of cell chemotaxis. (**a**) Microscopic images of migrated cells and non-migrated cells at 6 h and 12 h. Scale bar: 200 µm; Magnification: 200×. (**b**) Quantitative evaluation of cell chemotaxis, comparing the number of migrated and non-migrated cells or *CA* at 6 h and 12 h.

The migration process of cells in the microfluidic system is illustrated in Figure 5a. The upper and lower chambers are separated by a polyester carbonate membrane with uniformly distributed pores, facilitating cell passage. In the upper chamber, the cell suspension is injected and spread across the membrane. Cells adhere either individually or in clusters, displaying varied densities and morphologies, from round to stretched or spread, as they adapt to the microenvironment. Over time, cells migrate through the membrane pores into the lower chamber, accompanied by notable morphological changes. At 6 h, cells generally maintain a round shape with minimal deformation, indicating early-stage migration with limited environmental stress (Figure 5a). By 12 h, cells exhibit significant deformation, such as elongation or flattening, reflecting enhanced adaptability and migration efficiency. This deformation indicates that cells are making substantial adjustments to navigate the pores, overcoming environmental constraints, and improving their migratory capability. The increased deformation over time highlights how cells adapt to the mechanical stresses and spatial limitations of the microfluidic system [51].

Cell chemotaxis ability was quantified as shown in Figure 5b. At the 6 h mark, a notable increase in cell chemotaxis was observed, indicating that cells began actively responding to and adapting to the microfluidic environment. This initial rise is likely due to cell attachment in the upper chamber and their traversal through the polyester carbonate membrane pores. By 12 h, the number of migrated cells increased further compared to 6 h, suggesting continued migration and a larger accumulation of cells. The extended duration likely facilitated greater cell proliferation and spread within the microfluidic system. Factors influencing this trend may include cell–cell interactions, changes in the microenvironment, and the biological properties of the cells [52]. By 12 h, more favorable conditions, such as moderate intercellular gaps and increased migration space, likely contributed to the significant rise in migrated cell numbers.

### 3.4. Comparison of Different Methods

To evaluate the performance of the improved model, we conducted experiments using a sample of migrated cells, dividing it into training and test sets. The comparisons were made across several mainstream target detection networks, including SSD, Faster R-CNN, Yolov3-SPP, Yolov4, Yolov5, and the DCSPP-Yolo model. The results are summarized in Table 2.

The Yolo framework outperformed both SSD and Faster R-CNN. Notably, the DCSPP-Yolo model, as proposed in this study, exhibited superior precision and recall compared to existing YOLO models. Specifically, our model achieved precision and recall rates of 96.56% and 96.51%, respectively, which represent improvements of 2.33% and 4.86% over the Yolov5 model. These results demonstrate that the improved DCSPP-Yolo model efficiently and accurately identifies migrated and non-migrated cells.

**Figure 6:**
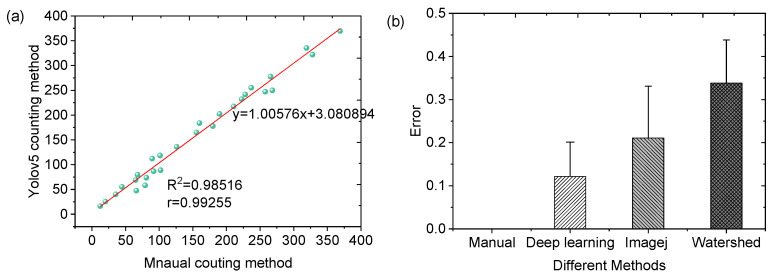
Validation of automated cell counting. (**a**) Relationship of migrated cell number obtained using the DCSPP-Yolo model and manual counting for the 12 h time point. (**b**) Comparison of cell-counting results according to different counting methods.

After identifying migrated and non-migrated cells, the number of migrated cells was quantified and compared with traditional counting methods. As shown in Figure 6a, the deep learning method demonstrated a strong correlation with manual counting, achieving an R^2^ value of 0.98. This high correlation underscores the superior accuracy of the deep learning method using microfluidic chip in cell counting.

In addition, compared to traditional manual counting and other automated methods, such as ImageJ 1.45 and Watershed, the deep learning approach improves the evaluation accuracy and reduces the identification error shown in Figure 6b. By minimizing counting errors, the deep learning method provides accurate quantification of cell chemotactic ability.

In addition to its accuracy and speed, the deep learning method also significantly reduces the overall time required for cell counting. As shown in Table 3, the total duration for the deep learning method in microfluidics is approximately 50 min, including ~30 min for dyeing and 20 min for fixing, which is considerably shorter than the 3–4 h required for manual counting.

Thus, it proves to be an effective tool for both accurately and efficiently counting migrated and non-migrated cells, ultimately realizing the rapid accurate evaluation of chemotaxis in microfluidic chips.

## 4. Conclusions

In this study, a microfluidic chip was successfully integrated with deep learning technology to improve the accuracy of identifying migrated and non-migrated cells, advancing our quantification of cell chemotactic ability. Initially, the microfluidic chip was designed, and experimental conditions were optimized, including chemoattractant concentration and migration time. In addition, a novel deep learning model was developed by incorporating a DCSPP module based on Yolo, introducing an attention mechanism and conducting a thorough analysis to identify optimal parameters. The improved model effectively identified and quantified migrated cells. The results show that this approach significantly reduces cell counting errors and provides a reliable means of evaluating cell chemotactic ability. The deep learning method in microfluidic chip demonstrates minimal error bars and exhibits stability and robustness across various experimental conditions.

However, there is potential for further improvement. Future research should focus on optimizing the model’s structure and parameters to enhance accuracy in identifying different cell chemotaxis states. Increasing the sample size is also recommended to validate the model’s applicability across a broader range of scenarios. Continued refinement is expected to offer more effective technical support for the use of microfluidic chips in biomedical research. Additionally, future studies could explore the development of real-time, non-destructive cell detection methods. Integrating real-time monitoring technology could provide a more comprehensive understanding of dynamic cell chemotaxis processes, leading to further optimization of deep learning models and enhancing the potential of microfluidic chips in cell research.

## Figures and Tables

**Table 1:** The optimal parameter combination of our algorithm.

Parameters	Optimal Choice
Batch size	16
Learning rate	0.01
Epoch	100
Activation function	Mish

**Table 2:** Comparison of different models.

Model	Precision Rate (%)	Recall Rate (%)
SSD	90.2	89.4
Faster R-CNN	91.56	88.87
Yolov3-spp	93.45	90.21
Yolov4	92.36	90.76
Yolov5	94.23	91.65
Our model	96.56	96.51

**Table 3:** Comparison of time duration of different methods.

Counting Method	Fixing Duration	Dyeing Duration	Drying Duration	Wiping Duration	Evaluation Duration	Total Duration
Deep learning in microfluidics		20 min			~30 min	50 min
Manual	30 min	20 min	10 min	3 min	2~3 h	3~4 h

## Data Availability

The original contributions presented in the study are included in the article, and further inquiries can be directed to the corresponding author.
